# Epidermoid cyst of the buccal mucosa—An uncommon entity: Case report and literature review

**DOI:** 10.1002/ccr3.4853

**Published:** 2021-09-21

**Authors:** Nouha Dammak, Abdellatif Chokri, Afef Slim, Ahlem Bellalah, Adel Bouguezzi, Sameh Sioud, Hajer Hentati, Jamil Selmi

**Affiliations:** ^1^ Oral Medicine Oral Surgery Department University Clinic of Dental Medicine Monastir Tunisia; ^2^ Oral Health and Oro‐Facial Rehabilitation Laborotary (LR12ES11) Faculty of Dental Medicine University of Monastir Monastir Tunisia; ^3^ Department of Pathology Fattouma Bourguiba University Hospital of Monastir Monastir Tunisia

**Keywords:** buccal mucosa, dermoid cyst, epidermoid cyst, oral cavity

## Abstract

Epidermoid cyst of the buccal mucosa is rare. Nevertheless, it must be included in the differential diagnosis of swelling in this area. The diagnosis is based on anatomopathological examination. The surgical enucleation is the gold standard of treatment.

## Graphical abstract

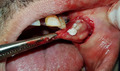

## 1 INTRODUCTION

Epidermoid, dermoid, and teratoid cysts are nonodontogenic benign lesions derived from the germinative epithelium, appearing any where of the body. In the oral cavity, these are uncommon and account for <0.01% of all the oral cysts. These cysts often remain asymptomatic for years however may become symptomatic as a result of enlargement or superinfection.

Epidermoid, dermoid, and teratoid cysts are nonodontogenic lesions derived from the germinative epithelium.^1^ These cysts can be found anywhere in the body, particularly in areas where embryonic elements fuse together.^2^, ^3^ Most of the reported cases have been localized in the ovaries, the testicles, as well as the hands and feet.^2^, ^3^ The incidence in the head and neck has been reported to be about 7%,^4^, ^5^ with only 1.6% of cases presenting in the oral cavity.^5^, ^6^ The diagnosis of epidermoid cyst remains a great challenge for clinicians; as the clinical aspect is not specific and may mimic many other diseases or conditions. Therefore, several investigations as ultrasonography, fine needle aspiration, and MRI are recommended to rule out other diagnosis.^7^, ^8^

The definitive diagnosis of epidermoid cyst is based on the anatomopathological examination. These cystic lesions were classified since 1955 by Meyer into epidermoid, dermoid, and teratoid variant. Dermoid cysts are lined by epidermis and contain skin adnexa such as sebaceous glands, sweat glands, and hair follicles. When there are no adnexa, these cysts are termed as epidermoid or epidermal with the lining containing only epithelium. Teratoid cysts consist of dermoid material plus tissue of other embryonal sources like respiratory, gastrointestinal, and connective tissues such as bundles of striated muscle and distinct areas of fat.^2^, ^3^

The midline or sublingual region of the mouth floor is the most commonly affected area contrary to the buccal mucosa which seems to be an unusual site of occurrence.^9^

## 2 CASE REPORT

A 56‐year‐old man with no past medical history was referred to the Oral Medicine and Oral Surgery department at the university clinic of dental medicine, Monastir, Tunisia, with the chief complaint of a painless swelling in the left buccal mucosa for about 5 years. The patient had neither known drug allergies nor history of surgery and/or trauma related to the lesion area.

**FIGURE 1:**
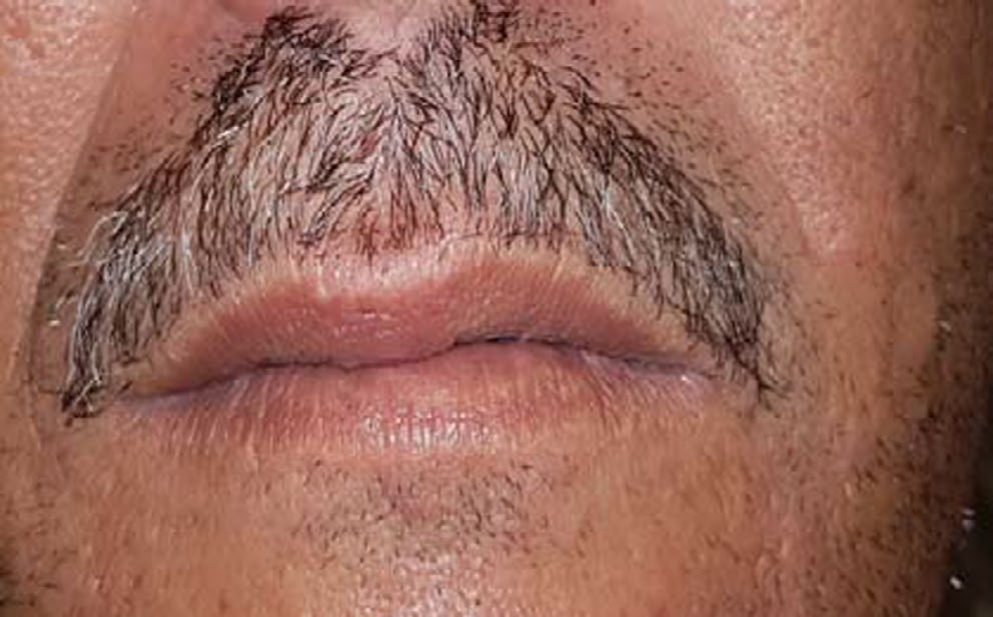
Extraoral view: swelling of the left cheek

Extraoral examination revealed the presence of a solitary swelling in the left cheek covered by normal looking skin in color and texture, as well. The swelling was nontender and freely mobile to the skin, on palpation. No submental nor submandibular lymphadenopathy was detected (Figure 1).

**FIGURE 2:**
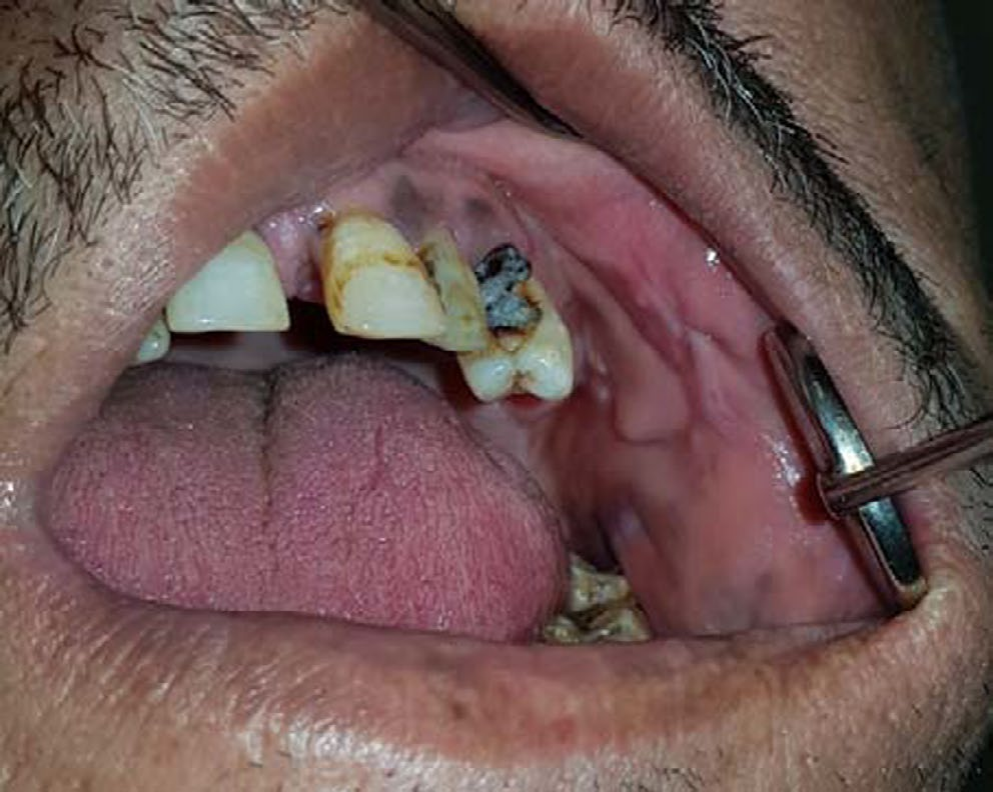
Intraoral view: a nodule in the left buccal mucosa covered by a healthy mucosa

Intraoral examination showed a poor oral hygiene and a nodule in the left buccal mucosa covered by a healthy mucosa. The mass was soft and non‐mobile on palpation (Figure 2).

Due to these clinical findings, the initial diagnosis was benign tumor of either the oral mucosa (including vascular lesions) or the salivary gland.

**FIGURE 3:**
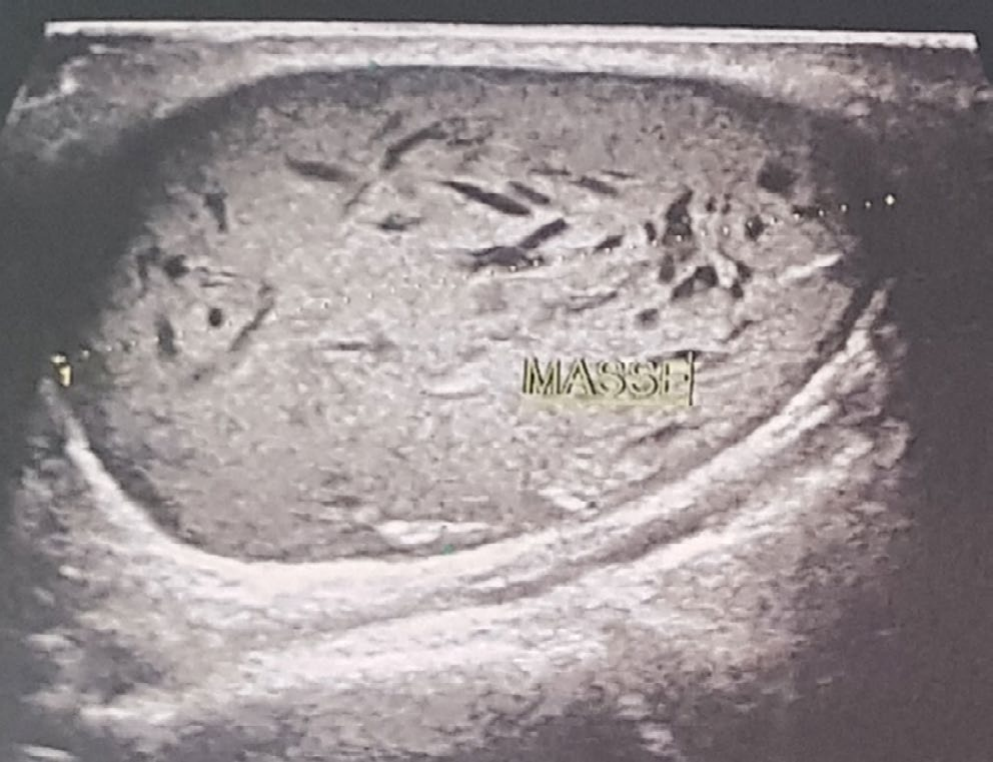
Echographic view: a well‐defined walled hyperechoic heterogenous lesion measuring 34 × 31 × 21 mm with posterior ultrasound reinforcement in the left buccal mucosa region

An ultrasonography was required. It revealed a well‐defined walled hyperechoic heterogenous lesion measuring 34 × 31 × 21 mm with posterior ultrasound reinforcement in the left buccal mucosa. Vascular lesions were excluded on Color Doppler analysis. At this stage, the diagnosis of epidermoid cyst of the left buccal mucosa was evoked (Figure 3).

**FIGURE 4:**
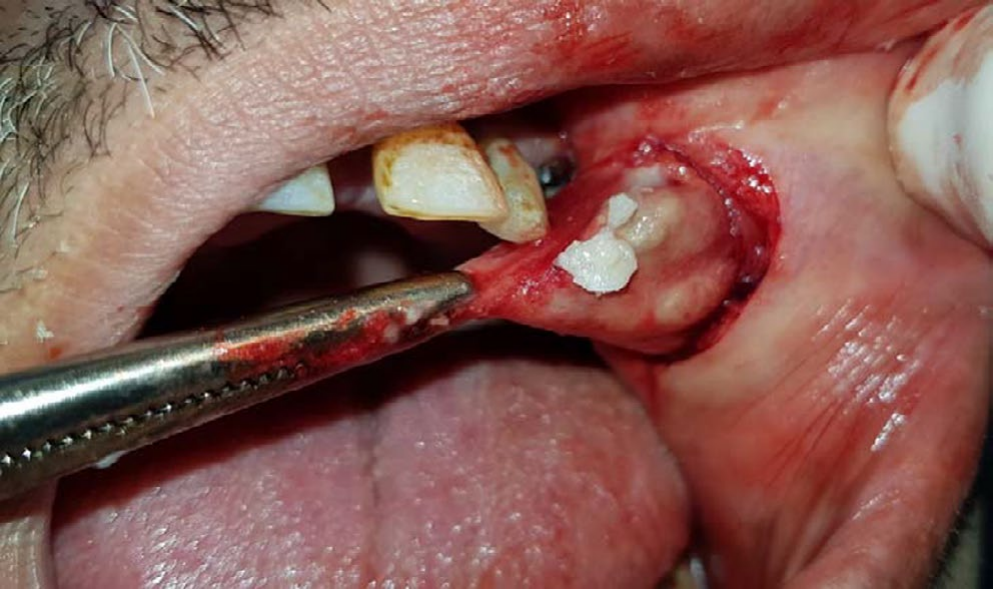
Peroperative view: cyst enucleation and the yellow color of the intra cystic liquid

**FIGURE 5:**
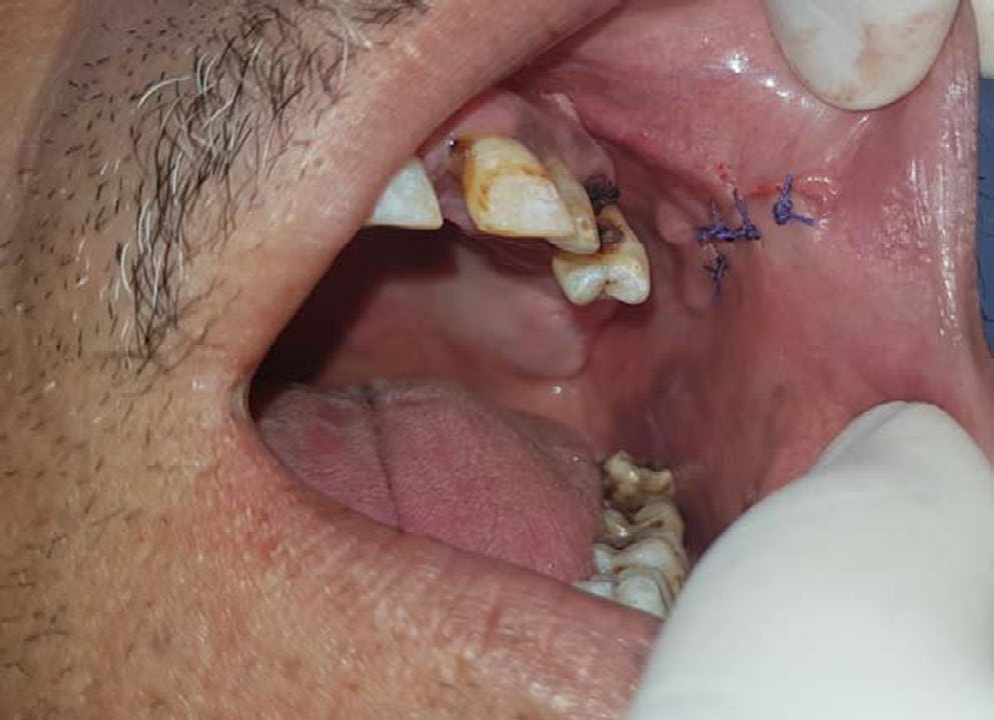
Postoperative view

Under local anesthesia and with intraoral access, the lesion was completely removed. An horizontal incision in the buccal mucosa was conducted exposing a cyst immediately underlying the mucosa. The enucleation was carefully done. Accidentally, when a partial rupture of the wall was happened, a yellow liquid was observed (Figure 4). The surgical wound was closed using a 4.0 silk suture, and the specimen was stored in 10% formol for further anatomopathological study (Figure 5). The postoperative period was uneventful and healing was good.

**FIGURE 6:**
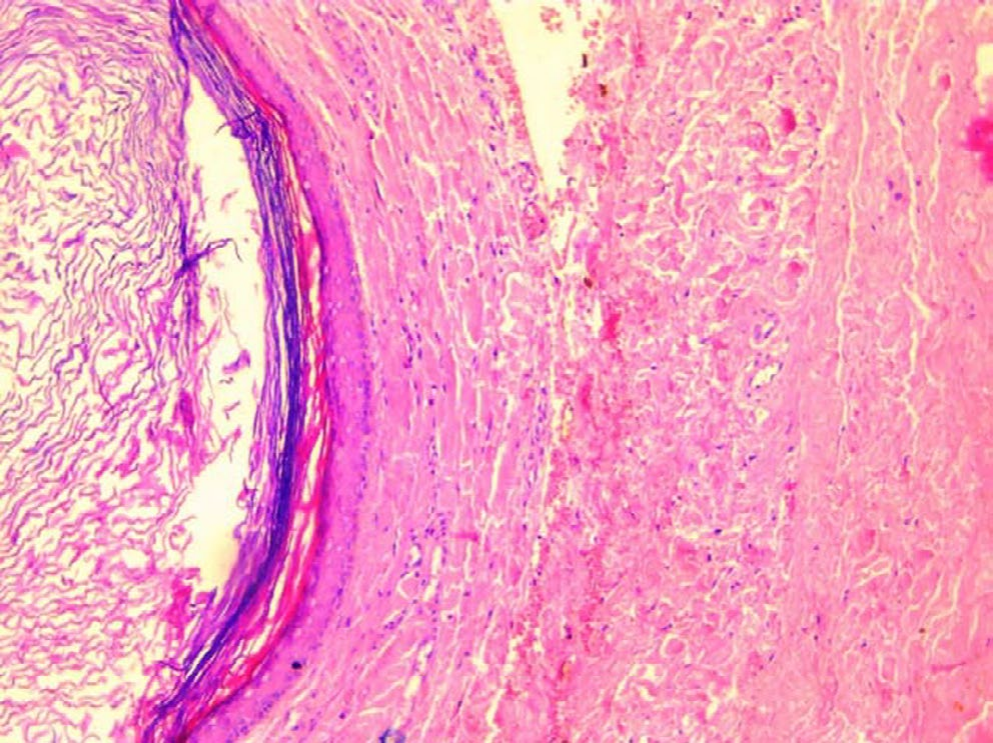
Cyst wall is composed of keratinized stratified squamous epithelium with keratin debris and no skin appendages

**FIGURE 7:**
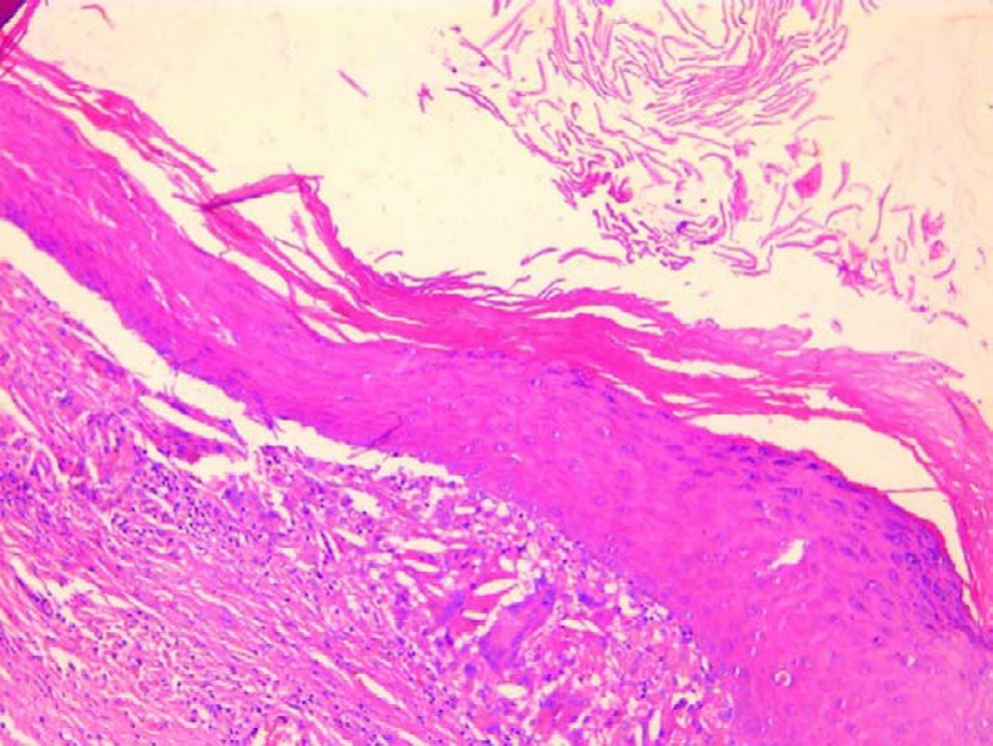
Cyst wall contains a foreign‐body giant‐cell reaction

Histopathological examination of the surgical specimen revealed a cystic cavity. The cyst wall is composed of keratinized stratified squamous epithelium with keratin debris and no skin appendages (Figure 6). This wall contains a foreign‐body giant‐cell reaction (Figure 7).

These findings were suggestive of epidermoid cyst.

The outcome was favorable after 1 year of follow‐up. No evidence of recurrence was recorded.

## 3 DISCUSSION

Dermoid cyst, epidermoid cyst, and teratoma are three histologically closed uncommon lesions englobed in the concept of dermoid cyst.^7^ These are nonodontogenic benign lesions. Dermoid cysts are found in the head and neck region in about 7% of all cases.^4^ The intraoral ones are rare and account for <0.01% of all the cysts.^2^, ^3^, ^4^, ^6^ The floor of the mouth is the most commonly affected area araising in 23% of the head and neck dermoid cyst,^10^ as the sublingual region is one of the sites of embryonic fusion.^4^

However, these cysts can also be found less frequently in the tongue, lips, uvula, jaw bones, and buccal mucosa.^3^, ^4^

The epidermoid cysts, which were firstly described by Rose in 1859,^1^ are the most common comprising 85–90% of all excised cysts.^9^, ^11^ Epidermoid cysts are also known by a number of other names, including follicular cysts, epidermal inclusion cysts, and wen.^2^, ^3^

A review of the literature was conducted on the database Medline via its interface PubMed using Mesh Keywords: “oral cavity,” “cyst, epidermoid,” “cyst, dermoid” and combining the following Boolean equations: “((cyst, epidermoid[MeSH Terms]) OR (cyst, dermoid[MeSH Terms])) AND (oral cavity[MeSH Terms]),” until December 2020. Moreover, the reference lists of relevant articles were manually searched for additional studies. This bibliographic research concluded to 9 case reports about epidermoid cyst of the buccal mucosa from 8 articles. The parameters extracted from these cases were summarized in Table 1 and concerned: gender, age, onset, site, and size.

**TABLE 1:** Review of the literature regarding epidermoid cysts arising in the buccal mucosa

Author	Year	Gender	Age (years)	Onset	Site	Size (cm)
Schneider^12^	1978	F	36	NI	Right	1
Schneider^12^	1978	F	30	3 years	Left	3
Gutmann^13^	1978	F	48	1 year	Right	1.5
Rajayogeswaran^15^	1989	M	25	1 year	Left	1.5*2
Ozan^3^	2007	F	38	6 months	Left	2*3*4
Kini^2^	2013	M	25	2 years	Left	1.5*1.5*1.5
Wildsom^9^	2015	M	29	4 years	Right	3.5
Srivastava^5^	2015	M	35	3 months	Bilateral	1.5*1.5
Rohde^8^	2019	F	2	2 years	Left	4.5 *3.5* 1.5
Present case	2021	M	56	5 years	Left	3.4*3.1*2.1

Note

F: female/M: male.

According to this literature review, the two first cases of epidermoid cyst in the buccal mucosa were published by Schneider, Mesa in 1978, and involved women in the fourth decade of their lives.^12^

In the same year (1978), Gutman et al.^13^ reported an atypical case of intradermal nevus which appeared to involve the wall of an epidermoid cyst. The authors believed that the cyst comprised the major portion of the lesion and originated independently of the associated nevus.

Epidemiologically, dermoid and epidermoid cysts may be present at birth and also in old patients, with the majority occurring in the second and third decades of life.^2^, ^10^ The mean age of included patients in this review was 29.77 years. The present case was a 56‐year‐old patient which seems to be an older age than that reported in most cases.

Even though that the frequency of occurrence of (epi)dermoid cysts is equal in both genders,^10^ Kim et al.^14^ reported that male were more affected than female, which was in agreement with the present case. Regarding the patients with epidermoid cysts of the buccal mucosa, including our case, 5 were male and 5 were female.

Many etiopathogenetic theories have been proposed for the development of (epi)dermoid cysts. These cysts are classified to congenital or acquired lesions.^2^, ^9^ Congenital cysts are dysembryogenetic lesions that arise from ectodermic elements, migrating into the facial midline, entrapped during the fusion of the first and second branchial arches between the 3^rd^ and 4^th^ weeks of intrauterine life.^3^, ^9^, ^15^ According to the theory of acquired development, the epidermis migrates into the deep tissue as a result of a physical trigger such as trauma or surgical complication and develops into an (epi)dermoid cyst.^16^ The posttraumatic cysts are also called as implantation keratinizing epidermoid cysts.^2^ Ozan et al.^3^ and Rajayogeswaran et al.^15^ do not believe in this congenital theory for the appearance of the lesion in the buccal mucosa. Posttraumatic cysts are usually asymptomatic and may not be associated in the patient's mind with any specific trauma, as this may be occurred several years earlier. For the present case, the patient is not sure if an injury occurred before the appearance of the lesion.

The size of the cyst is variable from millimeters till some centimeters, depending on its first clinical manifestation.^10^ This review reported that the size of epidermoid of the buccal mucosa is ranging from 10 mm to 40 mm.

There may be a left side predilection of the occurrence; as reported in the present case.

Swellings in the buccal mucosa may lead to a series of clinical diagnoses, as some conditions may present in a similar aspect making the diagnosis difficult.

Odontogenic infections affecting the facial spaces of buccinator and masseter muscles, pleomorphic adenoma, mucocele, cervical lymphadenopathy, hemangioma, lipoma, fibroma, neoplasms, and dermoid cyst are considered as differential diagnosis of lesions occurring in the buccal mucosa with a clinical aspect similar to the present case.^4^, ^9^, ^10^

In the present case, odontogenic infection was ruled out as the lesion was not associated clinical symptoms such fever and malaise. Such the lack of nodal involvement, the slow progression of the lesion and its benign appearance, neoplastic conditions were excluded, as well.

To achieve the correct diagnosis and differentiate between vascular, salivary and mucosal lesions, specialized imaging techniques such as ultrasonography (US), computed tomography (CT), magnetic resonance imaging (MRI) should be carried out.^4^ On CT scans, the dermoid cyst appears as moderately thin walled, unilocular masses filled with a homogeneous, hypoattenuating fluid substance with numerous hypoattenuating fat nodules giving the pathognomonic “sack‐ofmarbles” appearance.^4^ On MRI, the lesion appears as a well‐circumscribed mass. The signal intensity of epidermoid cysts is high in T2‐weighted images and low in T1‐weighted images. US is interesting such is a non‐invasive, easy, quick, and cheap test. US findings revealed a well‐circumscribed, smooth mass with a heterogeneous interior.^4^ In the present case, the US was useful in diagnosing the lesion on the buccal mucosa. Even the aspiration biopsy is commonly used; in many cases, it can result in a not reliable diagnostic sample.^10^ Thereby, imaging plays an important role, but the definitive diagnosis is based on the histopathological findings.

The surgical excision or enucleation is the gold standard treatment via an intraoral or extraoral approach, depending on the size and the location of the cysts.^10^ In all cases of this review, surgeries were performed. Recently, Mumtaz et al.^17^ reported the first case of dermoid cyst in the floor of the mouth being managed with marsupialization, as a good option of large epidermoid cysts.

Although recurrences have been described as <3%,^16^ these may be prevented by the complete removal of the cyst wall. There is no recurrence in all included cases.

Despite the benign nature and the good prognosis of dermoid or epidermoid cysts,^4^ isolated cases of premalignant and malignant conditions (Bowen's disease, Paget's disease, and squamous cell carcinoma) have been reported. Bhatt et al.^11^ described a case of a squamous cell carcinoma that appeared in the epithelium of an epidermoid cyst in the floor of the mouth, associated with the sublingual gland.

## 4 CONCLUSION

Epidermoid cyst of the buccal mucosa is extremely rare. Nevertheless, it must be included in the differential diagnosis of swelling in this area. To exclude other conditions, like benign tumors and odontogenic infection, appropriate imaging techniques are necessary in preoperative.

The diagnosis is based on anatomopathological examination. The surgical enucleation is the gold standard of treatment, and usually without recurrence.

## CONFLICTS OF INTEREST

None.

## AUTHOR CONTRIBUTIONS

ND wrote the manuscript. AC involved in surgery performance and manuscript revision. AS ensured patient follow‐up and involved in manuscript drafting. AB analyzed and interpreted the specimen. AB, SS, and HH involved in manuscript revision. JS revised the manuscript for important intellectual content.

## ETHICAL APPROVAL

This manuscript is the authors' own original work, which has not been previously published or considered for publication elsewhere. All authors have been personally and actively involved in substantial work leading to the paper and will take public responsibility for its content.

## CONSENT

The written consent for publication was released by the patient.

## Data Availability

The data that support the findings of this study are available from the corresponding author, upon reasonable request.
